# Supplementary material for: Identification of Genes Related to Squab Muscle Growth and Lipid Metabolism from Transcriptome Profiles of Breast Muscle and Liver in Domestic Pigeon (Columba livia)
Source: Animals (Basel). 2022 Apr 20;12(9):1061. doi: 10.3390/ani12091061 (PMC9100022; doi:10.3390/ani12091061)
Supplement: Supplementary file 1 [file animals-12-01061-s001.zip › Table S1. Primer sequences of the genes used in qRT-PCR testing.pdf]

**Table S1.** Primer sequences of the genes used in qRT-PCR testing.

| Gene | Forward prime (5′ → 3′) | Reverse primer (5′ → 3′) | Product size (bp) |
|---|---|---|---|
| *MYH7* | CCTCAACGAGACGGTGGT | TGATACAGCGGACGAAGTG | 221 |
| *FITM2* | AGCACCACCACTCACCTC | CCATCAGAACGCTCTTCG | 194 |
| *IRS2B* | AGAAACCAACCCAATCCT | TTGAATGTCATCTCCGTGT | 101 |
| *CD1D* | AACCTCATCTTATCCCTGTCC | GCTGCCAACTCACTCTTCTG | 203 |
| *CAMK1D* | GAGGGTAAAGGAGATGTGATG | AGCAGGATGTATGCGATG | 131 |
| *COL4A6* | TCCGTGCCACTCCGTTCA | CTGACAAACTGCTGCGACT | 112 |
| *SCUB1* | GCAAAGTGCCATTTACGG | GCAGCTTCTGCTCCATCC | 274 |
| *FABP3* | GGCTCACCAAACCCACCA | TGTCTCCTTCCCGTCCCAC | 206 |
| *MCM3* | GCGTGCCCTGAAGGATTT | GGACGACCTTTGGACGAAC | 197 |
| *CYP3A28* | CTGCCCAACAAGGCTACT | ATTGATCTCCACCGTCTG | 138 |
| *CLPS* | GCAGAGTCCCAGGATTGC | TCACTGTTGGTGACAGAGCC | 119 |
| *ACOX2* | AGGCTCACTGCCACTACAT | GAAGTCAAACGCATCCAC | 257 |
| *GFPT2* | ATTATGCCACTTGTCTGG | AAATGATTGGACGACCCT | 213 |
| *CCNB2* | AGAGGAGGATTTGTGCCA | CTTCCCGTCAAGGTAGTG | 175 |
| *CX3C* | TCATCTTGGTTGTGGTGGTC | GCAATGGTTTCGGTCAGG | 151 |
| *ACAP3* | ATTCTGCCACCGACTCCC | GAATGCCCAGGTTGATGC | 138 |
| *β-Actin* | GTGGATCAGCAAGCAGGAGT | TCATCACAAGGGTGTGGGTG | 101 |
